# Demographic and physiological signals of reproductive events in humpback whales on a southwest pacific breeding ground

**DOI:** 10.1093/conphys/coae038

**Published:** 2024-06-18

**Authors:** Logan J Pallin, Claire Garrigue, Nicholas M Kellar, C Scott Baker, Claire D Bonneville, Solène Derville, Ellen C Garland, Debbie Steel, Ari S Friedlaender

**Affiliations:** Department of Ecology and Evolutionary Biology, University of California Santa Cruz, Ocean Health Building, 115 McAllister Way, Santa Cruz, CA 95060, USA; Department of Ocean Sciences, University of California Santa Cruz, Ocean Health Building, 115 McAllister Way, Santa Cruz, CA 95060, USA; UMR ENTROPIE IRD, Université de La Réunion, Université de la Nouvelle-Calédonie, CNRS, IFREMER, Laboratoire d'excellence-CORAIL, 101 promenade Roger Laroque BP A5NOUMEA CEDEX5 Nouvelle Calédonie 98848, France; Opération Cétacés, BP 12827, Nouvelle-Calédonie 98802, France; Marine Mammal Turtle Division, , Southwest Fisheries Science Center, National Marine Fisheries Service, National Oceanic and Atmospheric Administration, 8901 La Jolla Shores Drive, La Jolla, CA 92037, USA; Department of Fisheries, Wildlife, and Conservation Sciences, Marine Mammal Institute, Oregon State University, Hatfield Marine Science Center, 2030 SE Marine Science Drive, Newport, OR 97365, USA; UMR ENTROPIE IRD, Université de La Réunion, Université de la Nouvelle-Calédonie, CNRS, IFREMER, Laboratoire d'excellence-CORAIL, 101 promenade Roger Laroque BP A5NOUMEA CEDEX5 Nouvelle Calédonie 98848, France; Opération Cétacés, BP 12827, Nouvelle-Calédonie 98802, France; UMR ENTROPIE IRD, Université de La Réunion, Université de la Nouvelle-Calédonie, CNRS, IFREMER, Laboratoire d'excellence-CORAIL, 101 promenade Roger Laroque BP A5NOUMEA CEDEX5 Nouvelle Calédonie 98848, France; Opération Cétacés, BP 12827, Nouvelle-Calédonie 98802, France; Sea Mammal Research Unit, Scottish Oceans Institute, School of Biology, University of St. Andrews, W Sands Rd, St Andrews KY16 9XL, UK; Department of Fisheries, Wildlife, and Conservation Sciences, Marine Mammal Institute, Oregon State University, Hatfield Marine Science Center, 2030 SE Marine Science Drive, Newport, OR 97365, USA; Department of Ocean Sciences, University of California Santa Cruz, Ocean Health Building, 115 McAllister Way, Santa Cruz, CA 95060, USA

**Keywords:** Biopsy, blubber, breeding ground, estradiol, estrous, humpback whale, progesterone, testosterone

## Abstract

The field of marine mammal conservation has dramatically benefited from the rapid advancement of methods to assess the reproductive physiology of individuals and populations from steroid hormones isolated from minimally invasive skin–blubber biopsy samples. Historically, this vital information was only available from complete anatomical and physiological investigations of samples collected during commercial or indigenous whaling. Humpback whales (*Megaptera novaeangliae*) are a migratory, cosmopolitan species that reproduce in warm, low-latitude breeding grounds. New Caledonia is seasonally visited by a small breeding sub-stock of humpback whales, forming part of the endangered Oceania subpopulation. To better understand the demographic and seasonal patterns of reproductive physiology in humpback whales, we quantified baseline measurements of reproductive hormones (progesterone—P4, testosterone—T and 17β-estradiol—E2) using an extensive archive of skin–blubber biopsy samples collected from female humpback whales in New Caledonia waters between 2016 and 2019 (*n* = 194). We observed significant differences in the P4, T and E2 concentrations across different demographic groups of female humpback whales, and we described some of the first evidence of the endocrine patterns of estrous in live free-ranging baleen whales. This study is fundamental in its methodological approach to a wild species that has a global distribution, with seasonally distinct life histories. This information will assist in monitoring, managing and conserving this population as global ecological changes continue to occur unhindered.

## Teaser

Assessing the reproductive physiology via skin–blubber biopsy samples of recovering populations of marine mammals is critical for conservation and management. We used an extensive blubber archive and associated demographic data to assess the seasonal changes in three reproductive hormones for humpback whales on a southwest Pacific breeding ground.

## Introduction

The field of marine mammal conservation has dramatically benefited from the rapid advancement of methods to assess the reproductive physiology of individuals and populations from steroid hormones isolated from minimally invasive skin–blubber biopsy samples (Hunt *et al*., 2013). Historically, this vital information was only available from complete anatomical and physiological investigations of samples collected from commercial or indigenous whaling or opportunistic stranding events (Lockyer, 1984). Sex steroid hormones are the main system-wide chemical signals of reproduction in mammals. As a result, these biochemicals have been increasingly used to help assess vital health and life-history states in populations that were previously depleted to historically low numbers (Hunt *et al*., 2013; Rocha *et al*., 2014; Clark et al., 2016; Pallin *et al*., 2018a; Pallin *et al*., 2023). Routine and long-term hormonal assessments facilitate continued monitoring of population growth and health in wild cetacean populations (Pallin *et al*., 2018a) and can inform wildlife managers of susceptible time periods in a species’ life history. Specifically, reproductive physiology is important for assessing density-dependent effects predicted for populations as they recover and approach pre-exploitation abundances or presumed carrying capacity (Baker and Clapham, 2004). This is particularly relevant for baleen whales, whose size makes captive studies largely impossible, and longitudinal observations and sampling of individuals in the wild are logistically challenging due to the environments in which they are found.

To better interpret these hormonal biomarkers, we need to combine them with information regarding both the life history and demography of the individuals being sampled across their entire seasonal life history. One particular area of research has focused on assessing the reproductive status of female cetaceans via the quantification of blubber steroid hormones (Clark, 2013; Kellar *et al*., 2013; Mello *et al*., 2017; Pallin *et al*., 2018a; Boggs-Russell *et al*., 2019; Dalle Luche *et al*., 2020). Three of the most common hormones used to assess reproduction include progesterone (P4), testosterone (T) and 17β-estradiol (E2). All three of these hormones are highly lipophilic and found in the ovaries (Pineda, 2003a). During the follicular phase, increased estrogenic (e.g. E2) secretion raises the blood supply to the uterus, resulting in growth and thickening of the endometrium (Pineda, 2003a). As a precursor to E2, T increases during the early follicular stage and may contribute to behavioural responses during estrous as well as having other functions (Pineda, 2003c). Following ovulation, progesterone levels increase as the corpus luteum develops in the ovary, signalling the myometrium to prepare for fertilization and implantation (Pineda, 2003a). Throughout gestation, progesterone aids in sustaining this environment for successful foetal development (Wuttke *et al*., 1998), returning to baseline levels prior to parturition (Bedford *et al*., 1972). As multiple hormones control the physiological mechanisms associated with reproduction, assessing a combination of these hormones and their ratios may allow for a more fine-scale understanding of temporal changes in reproduction.

The absence of hormonal baselines, particularly for a species like humpback whales (*Megaptera novaeangliae*) that exhibits two distinct seasonal behaviours (e.g. foraging, breeding/fasting), could result in the misinterpretation of the seasonality of blubber hormone levels. Previous studies investigating the relationship of sex steroid hormones in large whales have primarily focused on individuals sampled on migratory routes (Dalle Luche *et al*., 2020) or feeding grounds (Clark *et al*., 2016; Pallin *et al*., 2018a); however, to our knowledge, no information currently exists on the variation of blubber reproductive hormone concentrations during the breeding season. Since most reproductive processes are coordinated through hormones circulating in the bloodstream, and hormonal functions tend to be conserved across mammalian taxa. Endocrine analysis is a useful means to assess the reproductive status (e.g. pregnancy) and reproductive activity (e.g. estrous cycles, seasonality) of free-swimming individuals. Moreover, as steroids are highly lipophilic, the development of remote sampling techniques and methods to isolate and quantify several reproductive markers from skin–blubber biopsy samples allows us to better understand the full seasonality of reproductive hormones in humpback whales and potentially identify periods of time when populations may be more sensitive to disturbances during their annual breeding season.

Humpback whales are a migratory, cosmopolitan species that reproduce in warm, low-latitude breeding grounds and feed on high-latitude foraging grounds where they exploit dense aggregations of prey (Clapham, 2000). Located in the southwest Pacific, New Caledonia is seasonally visited by a small breeding sub-stock of humpback whales, forming part of the endangered Oceania subpopulation (Jackson *et al*., 2015). It is only in the last quarter-century; however, that this small breeding site has formally been described as both a calving and mating ground for Southern Hemisphere humpback whales due to continued observations of calves, as well as the acoustic detection of many males singing as part of courtship (Garrigue *et al*., 2001; Derville *et al*., 2019b). Peak mating within the South Lagoon occurs in August, with frequent sightings of whales occurring between June and September (Garrigue *et al*., 2001; Derville *et al*., 2019a). A long-term monitoring program (1993–present) has led to one of the largest and most comprehensive archives of skin–blubber remote biopsy samples collected for any Southern Hemisphere baleen whale population on their breeding ground.

The present study used skin–blubber biopsy samples collected from female humpback whales in New Caledonian waters between 2016 and 2019 to assess seasonal changes in reproductive hormones and set baselines for continued monitoring of this and other populations that are of conservation concern. The goal was to measure three reproductive hormones (P4, T, E2) to better understand the demographic and seasonal mechanisms of reproductive physiology in humpback whales. Our findings provide critical contextual data on how these hormones vary naturally in a population of humpback whales and support the continued use of minimally invasive skin–blubber biopsy samples to study reproductive physiology.

## Materials and Methods

### Sample collection

**Figure 1:**
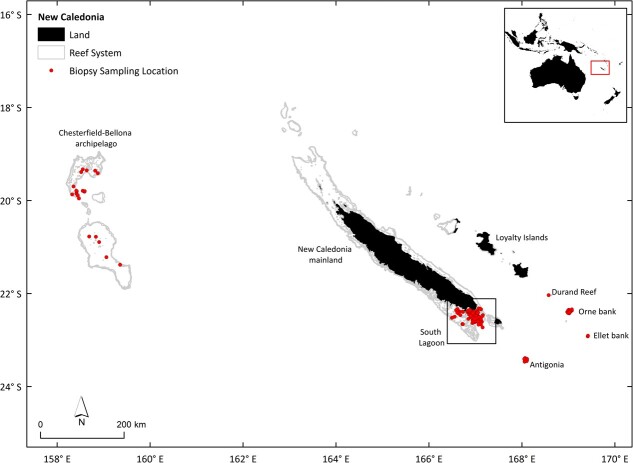
Study area and sampling locations of female humpback whales in New Caledonia from 2016 to 2019 used in the reproductive hormone analysis. Land is represented in black, and shallow reefs are represented in grey. The boxes delineate the south lagoon. Maps of land and coral reefs provided by Andréfouët *et al*. (2004).

Skin and blubber biopsy samples were collected from individual whales in New Caledonia (22°S, 166°E) during the austral winters (June–October) from 2016 to 2019 (Fig. 1) using standard field methods. Samples were primarily collected from the South Lagoon, the main coastal breeding area in New Caledonia (Derville *et al*., 2019b). Whales were approached with 6.30 m long semi-inflatable boats powered by two outboard engines (60 hp). For each group of whales encountered, the date, time, location (GPS position), group size and social context were recorded (Garrigue and Derville, 2022). The demographic and social context was noted, whether the whale was a singleton, member of a pair, mother–calf alone, mother–calf and escort or member of a competitive group. For competitive groups, the social role was specified including nuclear animal, mother, principal escort, challenger, secondary escort and unknown. The estimated age class of each individual whale was also recorded using three categories: adult, juvenile or calf (Garrigue and Derville, 2022). At the breeding grounds, calves are easily recognizable as they remain in close contact with their mother and reach a third to half of its length. (Clapham *et al*., 1999). Juveniles form an intermediate age-class (>1 year old), defining sexually immature whales that are smaller in size than a sexually mature, fully grown adult but larger than a calf. Juveniles have distinctive blurry patterns and colouring on the underside of their fluke and may sometimes display a curious attitude towards the boat (Garrigue and Derville, 2022).

Tissue samples were collected opportunistically from all age and sex classes, including calves, as allowed by annual research permits issued by the New Caledonian Government, which limits encounters of mother calf pairs to 1 hour/day (Garrigue and Derville, 2022). When mothers were biopsied, biopsy attempts were made on the calf first to prevent a possible reaction from the mother, which may either have terminated the encounter or made additional sampling more challenging (Garrigue and Derville, 2022). Samples were collected using either a crossbow with modified bolts (Lambertsen, 1987) and 40 mm tips (CetaDart) or a modified 0.22 calibre capture rifle with a detachable barrel and a valve to adjust pressure in the chamber (Paxarms, Cheviot, New Zealand; (Krützen *et al*., 2002)) deployed from the small vessel from a distance of 10–30 m targeting the area of the body in the vicinity of the dorsal fin. Darts used with the rifle were made from polycarbonate with stainless steel biopsy tips (6 mm diameter and 14–20 mm long) and were fired using a blank charge. Samples were stored whole on ice in the field and then frozen at −20^o^ C until used for analysis. Genomic DNA was extracted from the skin portion of the biopsy sample to identify sex (Gilson *et al*., 1998) and individuals via microsatellite genotyping (see Supplementary Material, Table S1) (Garrigue *et al*., 2004; Pallin *et al*., 2018a). When possible, photographs were collected to help identify animals based on the distinctive patterns of the underside of their caudal fluke or of their dorsal fin. Both photo-identification and genotyping allowed individual identification of whales sampled as part of this study. While sampling occurred opportunistically, it is important to note that sampling is weather-dependent, and, e.g. storms can greatly limit our ability to conduct adequate and consistent sampling of wild populations. As a result, it is important to mention that the demographic and seasonal comparisons made throughout this study are strictly a result of when the sampling was able to occur across the population.

### Hormone extraction and quantification

Steroid hormones were extracted from the blubber portion of the biopsy samples following standard methods (Kellar *et al*., 2006;Pallin *et al*., 2018a ; Pallin, 2022). Only female biopsy samples were analyzed as part of this study; however, we did analyze five male samples from 2019 as reference. These five samples were not included in any of the statistical analyses. Briefly, to quantify hormone biomarkers, we sub-sectioned a cross-sectional sample (~0.15 g) spanning from the epidermis–blubber interface to the most internal layer of the biopsy (~40 mm) to account for any depth variation in concentrations (Trana *et al*., 2015). These sub-samples were then homogenized multiple times using an automated bead mill homogenizer (Bead Ruptor Elite, Omni International) (Pallin, 2022). Following the completion of the homogenization process, we isolated three target hormones (P4, T, E2), using a series of 4:1 ethanol–acetone and ethyl ether washes and evaporations separations (Kellar *et al*., 2006; Pallin *et al*., 2018a). Lastly, target hormones were separated from the resulting lipid residue using a biphasic acetonitrile–hexane separation (Pallin *et al*., 2018a; Pallin, 2022). The final hormone residue was stored at −20^o^ C until analysis.

We quantified the amount of hormone in each blubber extract with commercially available enzyme immunoassays used extensively in similar studies (Riekkola *et al*., 2018; Pallin *et al*., 2018a; Pallin *et al*., 2018b; Pallin, 2022). The P4 EIA kit (EIA kit 900-011, ENZO Life Sciences, Farmingdale, NY, Enzo, 2014) used in our study had a 100% reactivity with P4 and an assay detection limit between 15 and 500 pg/ml. Two additional standard dilutions were added to allow for a lower detection limit of the standard curve to 3.81 pg/ml. The T EIA kit (EIA kit 900-065, ENZO Life Sciences, Farmingdale, NY, Enzo, 2015) used in our study had a 100% reactivity with T and an assay detection limit between 2000 and 7.81 pg/ml. Three additional standard dilutions were added to allow for a lower detection limit of the standard curve to 0.975 pg/ml. The E2 high sensitivity EIA kit (EIA kit 900-174, ENZO Life Sciences, Farmingdale, NY, Enzo, 2022) used in our study had a 100% reactivity with E2 and an assay detection limit between 15.6 and 1000 pg/ml. One additional standard dilution was added to allow for a lower detection limit of the standard curve to 7.8 pg/ml. All samples were run blind and in duplicate for all three hormones. For the assays, extracts were further diluted and re-run if concentrations fell beyond the detectability of the standard curve. Each assay was evaluated for colour development using a Biotek plate reader Epoch (Gen5™ software [Biotek, USA]) with reading and correction wavelengths of 405 nm and 630 nm, respectively. Blubber hormone concentrations were then transformed into nanograms of hormone per gram of blubber (wet mass).

As part of our routine quality control, we determined the extraction efficiency by spiking subsamples of blubber from a stranded, dead humpback whale with the target hormone (Kellar *et al*., 2006; Pallin *et al*., 2018a; Pallin, 2022) (150 ng of P4, 5 ng of T and 10 ng of E2). The percentage of each target hormone that was recovered after the extraction was calculated and each sample concentration was adjusted to this efficiency before statistical analyses (Pallin *et al*., 2018a; Pallin, 2022). An extraction efficiency greater than 60% was acceptable. If the extraction efficiency was less than 60%, the sample extracts were discarded, and the blubber samples were re-extracted. Additionally, we conducted parallelism and accuracy tests to gauge the performance of humpback blubber extracts with the T and E2 high sensitivity EIAK kits. This was done by taking a serially diluted pool of sample extracts and running them, along with the standard controls of the assay, to determine whether the linear decrease in measured values of the pooled sample was parallel to the standard curve. This would indicate that the assay measures the same antigens in the blubber as in the standards. Five extracts from five individual whales were pooled together for each assay, and the pooled sample concentrations were made by diluting five times from the neat preparation to 1/32, decreasing each time by a factor of two. Each dilution was run two times, and the resulting curve of the concentrations as a function of the mean optical density was compared to the standard curve. Progesterone parallelism for this species has been demonstrated by our research group previously (Pallin *et al*., 2018a) and has been extensively demonstrated elsewhere and as such was not repeated in this study (Clark *et al*., 2016; Mello *et al*., 2017).

### Data analysis

All statistical analyses were performed in RStudio (2023.06.1 Build 524) (R Core Team, 2021). We removed all within-year replicates from the data set to avoid re-sample bias in our analyses of demographic variation in reproductive hormones. In each case, the first sample was retained for the analysis. We tested differences in female reproductive hormones across demographic classes using an ANOVA and used a post hoc Tukey’s Honest Significant Difference test to check for specific differences among individual demographic groups. Correlations between steroid hormones and between the same hormones measured in mother–calf pairs were assessed using Pearson correlation. Yearly variation in female blubber hormone concentrations can be found in the Supplementary Material, Figure S1. Unless otherwise specified, all hormone values are reported as mean ± standard error (ng/g wet mass) and are also reported as per lipid mass in the supplemental table. We visualized seasonal patterns across ordinal days using hormone ratios and coloured lines were fit to data points using a glm model with ordinal day as a continuous covariate using the *ggplot2* package in R studio. The phenology of demographic groups was based on dates in which individuals were sampled. Progesterone concentrations were log transformed to reduce skewness and improve normality which was assessed via visualization of the Q–Q plot. We considered all statistical tests with a *P*-value of < .05 significant. All values are expressed as mean ± SD, unless otherwise stated.

### Animal ethics

This study was carried out following the marine mammal treatment guidelines of the Society for Marine Mammalogy. Fieldwork was undertaken under permits issued by the authorities of New Caledonia (provinces i.e.*.*, North N°s 60912-1247, -914, 60911-56 and South N°s 1105-2016, 899-2017, 2220-2018, and Government i.e*.* N°s 2016-1391, 2017-1107, 2018-1391, 2018-283, 2019-1291/GNC). The samples originating from outside U.S. jurisdiction were exported/imported under the Convention on International Trade in Endangered Species (CITES) permit numbers FR1998800067-E and 18US690343/9.

## Results

We analyzed 194 biopsy samples collected from female humpback whales (166 unique individuals, i.e. there were a total of 28 across season recaptures) in the waters of New Caledonia over the course of four field seasons from 2016 to 2019 (2016 *n* = 21, 2017 *n* = 84, 2018 *n* = 52, 2019 *n* = 37; Fig. 1). Efficacy of hormone quantification varied across the three different steroid hormones. E2 was quantified in all samples, followed by P4 (99.5%) and T (98.5%).

### Validation of humpback assays

**Figure 2:**
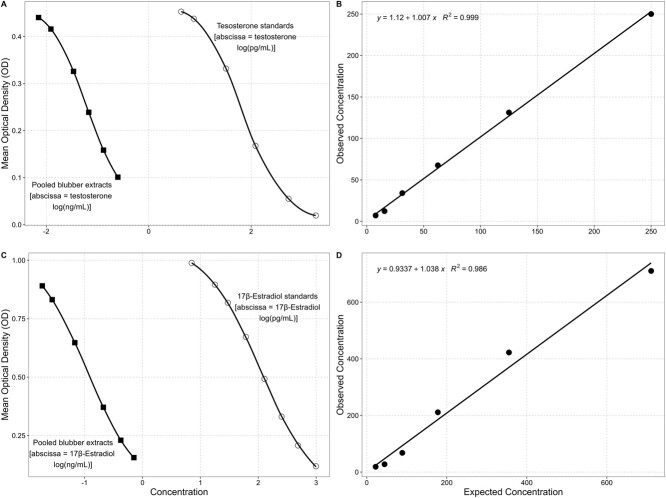
Enzyme immunoassay (EIA) validations for blubber testosterone (A, B) and blubber 17β-estradiol (C, D) extracted from blubber biopsy samples in humpback whales. Serial dilutions of extracts (shaded squares) showed strong parallelism with the standards (open circles) of the testosterone (A) and 17β-estradiol (C) EIA. Good accuracy was demonstrated by the positive linear relationship found in both assays. Testosterone (B) concentrations against apparent concentrations in samples (*R*^2^ = 0.999, slope = 1.007) and 17β-estradiol (D) concentrations against apparent concentrations in samples (*R*^2^ = 0.986, slope = 1.038). Both tests indicate that each assay is measuring the same antigens in the blubber as in the standards and is therefore suitable for use with humpback whale blubber tissues extracts.

Based on the concentrations observed from a series of spiked controls, our average extraction efficiency for P4 was 94.84% ± 11.13 (minimum 69.06%, maximum 93.35%), T was 80.09% ± 11.18 (minimum 63.68%, maximum 89.81%) and E2 was 82.41% ± 19.94 (minimum 68.12%, maximum 90.29%). The EIA standards and the pooled serially diluted blubber extracts for T (Fig. 2A and B) and E2 (Fig. 2C and D) exhibited statistical parallelism and high accuracy (T: *R*^2^ = 0.999, slope = 1.007; E2: *R*^2^ = 0.986, slope = 1.038); an indication that the assays were measuring the same antigens in the blubber as in the standards and are, therefore, suitable for use with humpback whale blubber tissues extracts. The calculated intra-assay and inter-assay %CV from a series of replicated samples were 3.39% and 7.21% for P4, 8.79% and 11.82% for T and 8.81% and 10.80% for E2. These results are consistent with previous studies on humpback whales (Mingramm *et al*., 2020).

### Demographic variation in reproductive hormones

**Figure 3:**
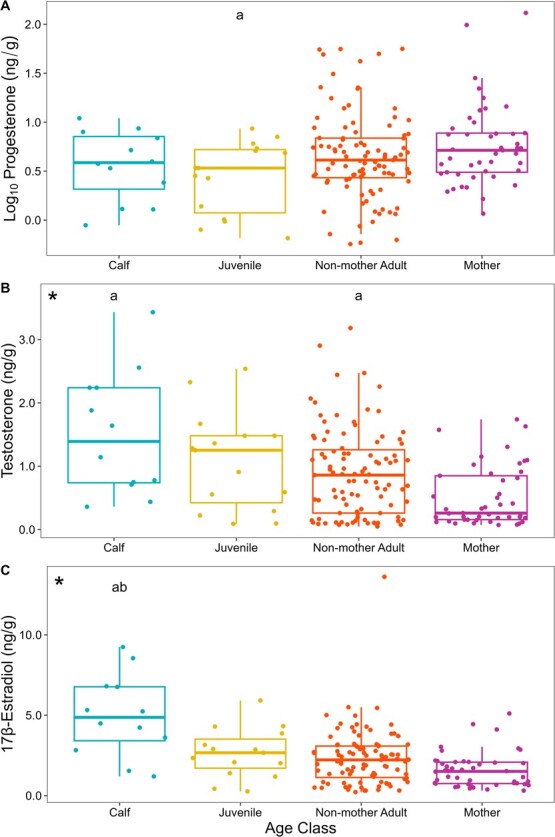
Demographic variation in blubber progesterone (A), testosterone (B), and 17β-estradiol (C) concentrations (ng/g wet mass) of humpback whales sampled in New Caledonian waters from 2016 to 2019. The asterisk (*) denotes a comparative statistically significant result via ANOVA. *Post hoc* multiple comparison analysis: a-significantly different from “Mother”; b-significantly different from “Non-mother Adult.”

Demographic variation in hormone concentrations across all 194 female humpback whale biopsy samples can be found in Table 1. We observed no significant differences in the P4 (*r*^2^ = 0.027, *F*_3167_ = 2.549, *P =* 0.058) concentrations, but observed significant differences in the T (*r*^2^ = 0.089, *F*_3167_ = 6.466, *P < 0.001*) and E2 (*r*^2^ = 0.115, *F*_3167_ = 8.429, *P < 0.001*) concentrations among the different demographic groups (Fig. 3). Calves had both the highest T and E2 concentrations across all demographic groups. A *post hoc* multiple comparison analysis is depicted in Fig. 3.

**Table 1:** Blubber sex steroid concentrations (mean ± SD ng/g wet mass) in biopsied humpback whales in New Caledonia from 2016 to 2019

Steroid hormone	Demographic class	Hormone concentration (ng/g wet mass)
Progesterone	All female samples (*n* = 193)	8.76 ± 14.87
	Calf (*n* = 12)	5.09 ± 3.74
	Juvenile (*n* = 15)	3.62 ± 2.49
	Non-mother adult (*n* = 96)	8.30 ± 12.39
	Mother (*n* = 42)	11.72 ± 24.23
	Male (*n* = 5)	2.16 ± 1.33
Testosterone	All female samples (*n* = 191)	0.86 ± 0.72
	Calf (*n* = 12)	1.58 ± 1.05
	Juvenile (*n* = 15)	1.08 ± 0.77
	Non-mother adult (*n* = 96)	0.89 ± 0.69
	Mother (*n* = 42)	0.54 ± 0.48
	Male (*n* = 5)	1.98 ± 1.86
17β-estradiol	All female samples (*n* = 194)	2.32 ± 1.85
	Calf (*n* = 12)	5.07 ± 2.69
	Juvenile (*n* = 15)	2.65 ± 1.54
	Non-mother adult (*n* = 96)	2.32 ± 1.76
	Mother (*n* = 43)	1.65 ± 1.1
	Male (*n* = 5)	0.56 ± 0.24

For the demographic class, “All Female Samples” includes all samples, including within year recatpures (n = 194), while “Calf”, “Juvenile” … etc. only includes the number of unique individuals annually (e.g. within year recatpures are removed, n = 166). Note; “all female samples” for progesterone has only 193 samples as one sample from a failed to provide any concentration and testosterone only has 191 samples as three samples failed to provide any concentrations. The total number of unique individuals for progesterone and testosterone is 165, as one individual sample failed to produce any concentration.

**Figure 4:**
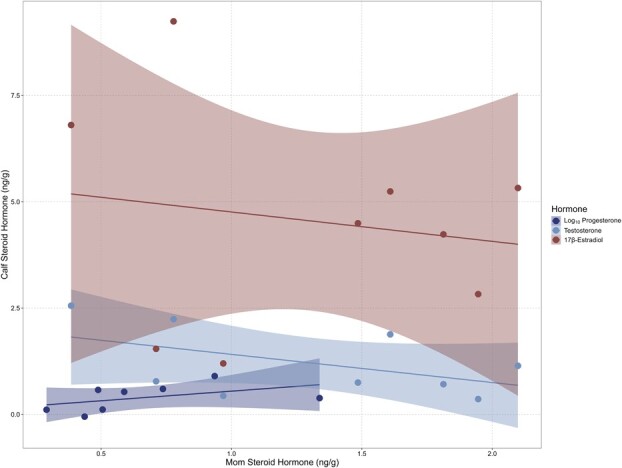
Blubber steroid hormone (progesterone, testosterone, 17β-estradiol) correlations of female humpback whale mother-calf pairs sampled in New Caledonian waters between 2016 and 2019. Note: one pair did not have measurable progesterone concentrations.

We sampled a total of nine mother–calf pairs (one pair did not have a complete set of progesterone values) over the course of this study and found no significant relationships between their paired hormone concentrations (P4: *r* = 0.476, 95% CI: −0.344 to 0.884, *P =* 0.233; T: *r* = −0.500, 95% CI: −0.874 to 0.246, *P =* 0.171; E2: *r* = −0.167, 95% CI: −0.748 to 0.559, *P =* 0.667; Fig. 4).

### Seasonal variation in reproductive hormones

**Figure 5:**
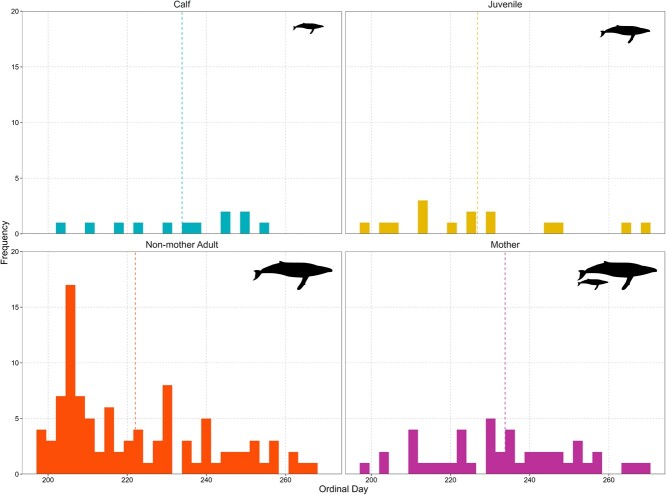
Encounter frequency histograms from biopsy sampling events of different demographic groups of female humpback whales sampled in New Caledonian waters between 2016 and 2019. The vertical dashed line represents the distribution mean. Humpback whale silhouette was created by Chris Huh, and retrieved from the Phylopic dataset (Keesey, 2023) under a CC BY-SA 3.0 DEED license (https://creativecommons.org/licenses/by-sa/3.0/).

**Figure 6:**
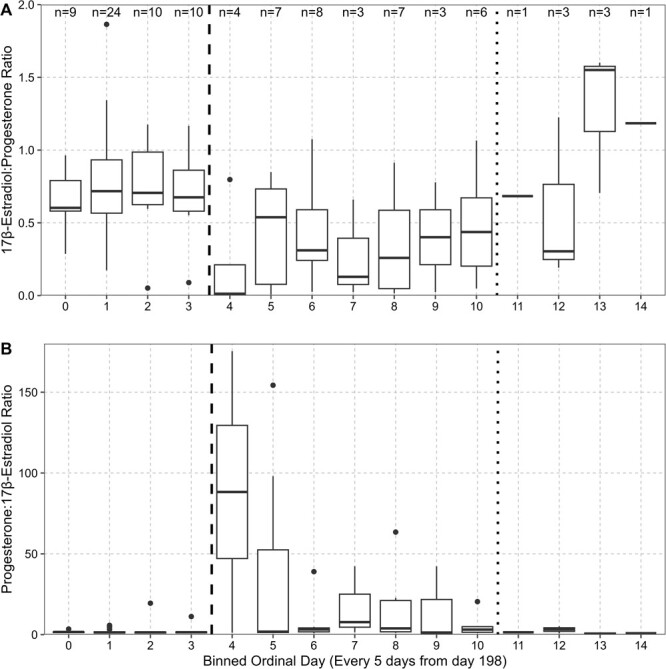
(A) Blubber 17β-estradiol and progesterone ratios in non-mother adult female humpback whales, binned in 5-day increments starting from ordinal day 198 (July 16). (B) Blubber progesterone and 17β-estradiol ratios in adult, non-mother female humpback whales, binned in 5-day increments starting from ordinal day 198 (July 16). The dashed black line represents end of early sampling season, the dotted line represents beginning of late sampling season, and between the two lines is the middle of the sampling season. Sample sizes are displayed in panel 6A.

**Figure 7:**
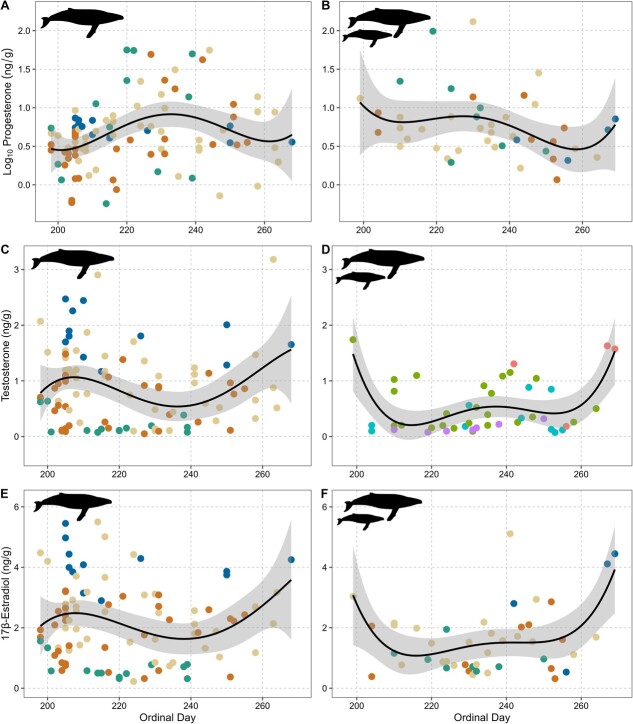
Seasonal blubber progesterone (A, B), testosterone (C, D), and 17β-estradiol (E, F) concentrations based on sampling day in non-mother (left column; n = 99) and mother (right column, n = 46) adult female humpback whales sampled in New Caledonian waters between 2016 and 2019. Grey-shaded areas represent the 95% confidence level interval. The Humpback whale silhouette was created by Chris Huh and retrieved from the Phylopic dataset (Keesey, 2023) under a CC BY-SA 3.0 DEED license (https://creativecommons.org/licenses/by-sa/3.0/).

Across the 4 years of this study, non-mother adults tended to be most abundant earlier in the sampling season (peak ordinal day: 205, average ordinal day: 222, August 10), followed by juvenile females (average ordinal day: 226, August 14) and lastly mothers and their calves (average ordinal day: 233, August 21; Fig. 5). The early sampling season (first 20 days; July 16–Aug 7) was dominated by higher E2/P4 ratios. This was followed by a flipped ratio of higher P4/E2 concentrations in the middle of the season, then by a second wave of higher E2/P4 ratios (Figs 6 and 7) later in the season.

### Correlation analysis of hormone pairs in female humpback whales

**Figure 8:**
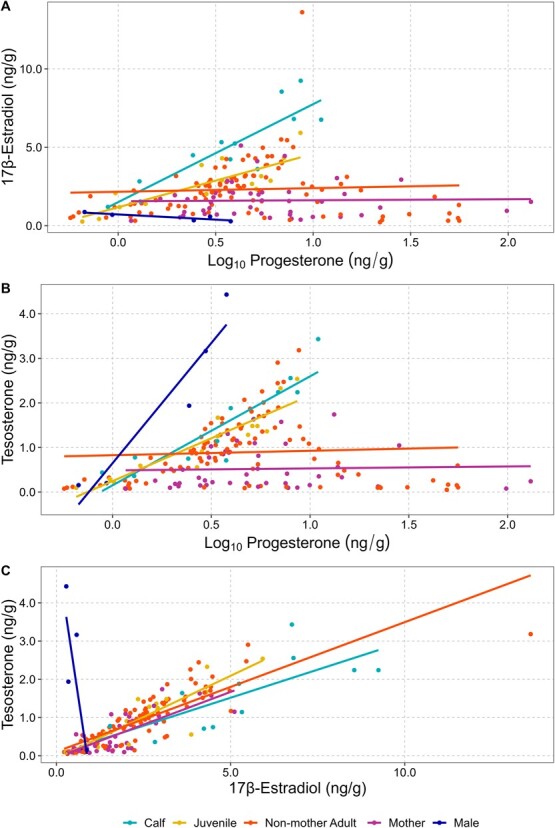
Correlations between blubber steroid pairs (A: progesterone to 17β-estradiol, B: progesterone to testosterone, C: 17β-estradiol to testosterone) among different female demographic groups of humpback whales sampled in New Caledonia waters between 2016 and 2019. Note: the five males are included as reference and not included in the statistical analyses.

P4 and E2 were not correlated among female humpback whales classified as non-mother adult (*r* = 0.059, 95% CI: −0.140 to 0.254, *P =* 0.562) and mothers (*r* = 0.024, 95% CI: −0.271 to 0.316, *P =* 0.874; Fig. 8), but were strongly correlated among juvenile (*r* = 0.819, 95% CI: 0.529–0.938, *P <* 0.001) and calf humpback whales (*r* = 0.878, 95% CI: 0.612–0.965, *P* < 0.001*.* Similarly, P4 and T were not correlated among female humpback whales classified as non-mother adult (*r* = 0.064, *P =* 0.530) and mothers (*r* = 0.039, *P =* 0.804; Fig. 8), but were strongly correlated among juvenile (*r* = 0.923, 95% CI: 0.779–0.974, *P* < 0.001) and calf humpback whales (*r* = 0.899, 95% CI: 0.72–0.972, *P* < 0.001. Conversely, E2 and T were strongly correlated in all demographic groups of female humpback whales (non-mother adult: *r* = 0.858, 95% CI: 0.795–0.902, *P <* 0.001; mothers: *r* = 0.794, 95% CI: 0.653–0.882, *P* < 0.001; juvenile: *r* = 0.861, 95% CI: 0.625–0.983, *P <* 0.001; calf: *r* = 0.768, 95% CI: 0.348–0.931, *P* = 0.004; Fig. 8). Hormone concentrations from the five control males are included as reference in Fig. 8.

## Discussion

The quantification and seasonal rates of change of reproductive hormones in cetaceans can provide valuable information about the reproductive parameters and reproductive potential of wild populations. To our knowledge, this is one of the first investigations into the seasonal and demographic variation in reproductive hormones of humpback whales on a Southern Hemisphere breeding ground. Such information provides critical contextual data on how these hormones vary naturally across a breeding season in a population of humpback whales and provides a basis for future comparisons. This is especially relevant as different demographic groups may have different susceptibilities to perturbations in their environment.

### Demographics-first principles

We observed significant differences in steroid hormone concentrations across different demographic groups of female humpback whales. The reproductive cycle in therian mammals involves recurring physiological changes induced by several reproductive hormones. During the follicular phase of the estrous cycle the rapid growth of a dominant ovarian follicle increases oestrogen (i.e. E2) production preparing the endometrium for implantation (Pineda, 2003a). As well, these high levels of E2 trigger the hypothalamus to release gonadotropin-releasing hormone (GnRH), causing the subsequent release of follicle-stimulating hormone and luteinizing hormone (LH) by the pituitary gland (Pineda, 2003a). This surge in LH results in the rupture of the dominant ovarian follicle, releasing the egg, and the remains of the follicle develop into the corpus luteum (CL) and begin to produce P4 (Pineda, 2003a). In studied captive cetaceans, under normal conditions, the CL remains active during the entire duration of the pregnancy, sustaining elevated levels of progesterone, which are necessary for the establishment and maintenance of the pregnancy (Robeck and O'Brien, 2018). Shortly following parturition or in the event the egg is not fertilized, the CL degenerates relatively rapidly into a non-functional body, the corpus albicans and progesterone levels return to baseline (Robeck and O'Brien, 2018). As well, we suspect that T is largely acting as a pre-cursor to estrogens in the biosynthesis of E2, as supported by our observed relationships between T and E2 in all sampled female whales and no apparent relationship in the reference males, but also has additional roles in the sexual development of younger females and the reproductive behaviour and physiology of adult females (e.g. receptivity of breeding events) (Pineda, 2003a). In this study, adult female humpback whales classified as “mothers” had the highest P4 concentrations. The goal of this study was not to detect pregnancy but to describe the signals of reproductive hormones during the breeding season. As a result, one confounding factor within the detected high P4 values among those individuals classified as ‘adults’ or ‘mothers’ is whether the high blubber P4 concentrations are attributed to either one, recent parturition or two, recent conception. If we assume that all females with calves will not get pregnant, then we would suspect the high P4 values would be attributed to a recent parturition instead of a recent ovulation (Browning *et al*., 2009). In this scenario, however, we cannot account for undetected calf mortality and perinatal losses that occurred prior to the encounter of the adult female, leading us to assume the high P4 values are more likely attributed to a recent ovulation. In killer whales, circulating P4 concentrations among pregnant females only started to significantly differentiate from non-pregnant females after the third-week post-conception (Robeck *et al*., 2016). Also, blubber cortisol concentrations have been shown to double within hours of controlled exposure experiments in captive delphinids (Champagne *et al*., 2018). However, we still have little to no understanding of hormone augmentation and degradation times in the blubber tissues of large whales, largely due to the infeasibility of captive and routine studies.

We found significant relationships between T and E2 in all sampled female humpback whales. We hypothesize that this relationship results from the steroidogenic pathway leading to the biosynthesis of E2. T may be converted to E2 within the blubber by the enzyme aromatase (P450_aro_, CYP19), an enzyme found in lipid-rich adipose tissues, like blubber in marine mammals, which actively converts T to E (Zhao *et al*., 1995; Norris and Carr, 2020). As mentioned briefly above, three reference males in our study with high T blubber concentrations had no elevated E2 concentrations. We believe this is a result of E2 not playing a significant role in male reproductive physiology (Pineda, 2003b).

Female humpback whale calves had the highest E2 and T concentrations of any demographic group. Evidence suggests that during late pregnancy, the production of E2 may be of foetal–placental origin (Ash and Heap, 1975; Hoffmann *et al*., 1976; Robeck *et al*., 2016). For example, in captive killer whales, a 33-fold increase in serum E was found within the umbilical cord compared to circulating levels in the mother (Robeck *et al*., 2016). This was followed by a rapid decline in oestrogen concentrations to baseline levels within three days after birth. While the high E2 values in calves could be residual carryover from gestation, we speculate that this observation is more directly related to sexual development (e.g. determination of biological sex and secondary sexual characteristics), neural development and maturation in pre-pubertal individuals (Day *et al*., 1984; Pineda, 2003a; Cardoso *et al*., 2020). Attainment of puberty requires full development of the reproductive neuroendocrine axis and subsequent release of high-frequency, episodic pulses of GnRH and LH, allowing the female gonads to become capable of releasing gametes (Pineda, 2003a; Cardoso *et al*., 2020). As a result, the key limiting physiological factor for the attainment of puberty would be the lack of these frequent pulses of GnRH and LH. This mechanism is maintained primarily through a negative feedback loop involving E2 (Cardoso *et al*., 2020). Our dataset further supports this as female humpback whales classified as juveniles had the second highest E2 concentrations. While juvenile female humpback whales are likely biologically “imprinted” to start reproduction on the breeding grounds, they may still be undergoing strong sexual development, resulting in the need to maintain the negative feedback system involving E2 (Cardoso *et al*., 2020). The complementary high concentrations of T in both calves and juveniles are likely a result of the role T plays in the biosynthesis of E2, as noted above (Pineda, 2003a). Additional monitoring, including epigenetic assessment of ageing, may help us better understand this transition from pre-pubertal to sexual maturity in humpback whales.

Necessary for the discussion of endocrine patterns between demographic groups is that the designation of age classes, i.e. ‘juvenile’, were assigned based on the best guess of the field team during field operations. Across all three hormones, there were clusters of ‘adults’ with low hormone concentrations. Puberty or attainment of sexual maturity is defined as the ability to accomplish reproduction successfully and should not be considered a single event but rather a process that occurs over time (Senger, 2012). The age at first estrous is the period in which a female becomes sexually receptive. In many animals, this often involves outward behavioural signs of sexual receptivity, especially in the presence of a male (Senger, 2012). However, the actual age at which a female can fully support her first pregnancy without adverse effects may come a few years after her first estrous cycle (Senger, 2012). The breeding system and behaviour of humpback whales generally consists of intense levels of male–male competition (e.g. competitive group) for a female (Clapham, 1992). Female humpback whales, on average, reach sexual maturity (i.e. puberty) between 4 and 6 years of age (Chittleborough, 1955; Clapham, 1992), and studies in southeastern Alaska have revealed ages at first calving in known humpback females between 8 and16 (average 11.8) years of age (Gabriele *et al*., 2007; Best, 2020). This discrepancy in age between when cycling begins and when the first pregnancy occurs may be related to these low hormone values in ‘adult’ whales. Simply, these females may undergo the behavioural displays of estrous without being pregnant.

### Demographic distributions

Throughout this study, non-mother adult female humpback whales were the first to arrive on the breeding ground, followed by juveniles, and lastly, mothers and their calves. These results corroborate prior observations of the temporal segregation of humpback whales in the Southern Hemisphere based on demographic status (Dawbin, 1966; Dawbin, 1997; Pallin *et al.*, 2018a). Dawbin (1966) reported that lactating females (i.e. females weaning yearlings from the previous breeding season which would not considered mothers in this study as the calf is now nearly 1-year-old) were the first whales observed to be migrating north near Cook Strait, New Zealand, followed by immature juvenile humpback, then males with resting females and finally pregnant females about to give birth (i.e. mothers in the context of this study).

### Oestrogen–Progesterone ratios and estrous cycles

This study described some of the first endocrine evidence consistent with estrous in a live baleen whale. E2:P4 ratios across adult female humpback whales were highest in the first 20 days of the breeding season, followed by an increase in P4:E2 ratios, and lastly, a second peak in E2:P4 ratios at the tail end of the season. Estrogens (e.g. E2) are associated with having multiple functions during estrous and pregnancy in different species (Ishikawa *et al*., 2004; Robeck *et al*., 2016; Norris and Carr, 2020). Post-conceptive increases in E2 are critical for the maternal recognition of pregnancy in pigs, and early elevations of E2 are associated with implantation in primates (Robeck *et al*., 2016). As a result, E2 can be a critical indicator of ovarian activity and sexual maturity in female mammals (Robeck *et al*., 2009; Senger, 2012). Seasonal variations, including an increase in serum E2 concentrations, have been correlated with estrous and implantation in other marine mammals, including California sea lions (*Zalophus californianus*) (Greig *et al*., 2007) and Pacific white-sided dolphins (*Lagenorhynchus obliquidens*) (Robeck *et al*., 2009). Additionally, as mentioned briefly above, P4:E2 ratios would increase during the luteal phase following ovulation (Robeck *et al*., 2016). Chittleborough (1954) concluded that female humpback whales are seasonally polyestrous, with estrous occurring on the Australian breeding grounds from June to October (Matthews, 1937), with a peak in ovulation in mid-July, continuing on a lessened scale through early October. Based on this information, the high E2:P4 ratios during the early part of the breeding season are consistent with pre-ovulatory follicular development and ovulation (e.g. period of receptivity), followed by the luteal phase (high P4 values) across the population (Cardoso *et al*., 2020).

The second peak in E2:P4 ratios at the end of the season are consistent with late arriving and late ovulating females or females undergoing a second ovulation. Chittleborough (1954) showed that a minimum of sixteen percent of humpback females had ovulated twice, with a second peak in ovulations in mid-September and Robins (1954) found evidence of multiple ovulations following unsuccessful fertilization. This was further supported by signs of rapid CL regression with no signs of a prolonged CL (Robins, 1954). Seasonal polyestrous cycles have also been observed in Pacific white-sided dolphins (Robeck *et al*., 2009), Indo-Pacific humpback dolphin (*Sousa chinensis*)(Brook *et al*., 2004) and the Atlantic bottlenose dolphin (*Tursiops truncatus*) (Robeck *et al*., 2005). It is important to note that in whales, these concepts are still based on the examinations of carcasses from over 60 years ago when population numbers were much lower due to commercial whaling and climate conditions looked very different. As a result, the total number of both seasonal ovulations and ovulations over the 1–3-year hypothesized breeding cycle warrants further research. Thus, continued analysis of sample archives and sampling of adult female humpback whales on the breeding ground, including serial sampling of the same individual across and within a season, is needed to better understand these seasonal reproductive dynamics.

### Conservation implications

In Cetartiodactyla (cetaceans and artiodactyls), several stressors have been shown to affect all main aspects of reproductive endocrine activity, leading to irregular estrous cycles, implantation failure, spontaneous abortion and elevated infant mortality (Hennessy and Williamson, 1983; Moberg, 1991; Wilson *et al*., 1998) In cetaceans specifically, the cumulative effect of changing behaviours, displacement or chronic stress induced by exposure to whale-watching activity has been linked to a decline in female reproduction (Bejder *et al*., 2006a; Bejder *et al*., 2006b). New Caledonia is among the leading countries for humpback whale watching in Oceania, and a study conducted in 2013 showed that over 80% of humpback whales approached by boats in the region significantly changed their behaviour (Schaffar *et al*., 2013). Here, we have presented evidence consistent with ongoing estrous at the population level in female humpback whales breeding within New Caledonia waters, indicated by high E2 relative to P4 early in the season. Exposure to stressors during this window in a female humpback whale’s life history may negatively affect her reproductive physiology. Across the population, this may result in adverse population-level effects. We encourage adaptive management within this sub-stock of humpback whales to mitigate any potential negative impacts on whale reproductive physiology (e.g. limit disturbance during periods of peak estrous) so that we can ensure this population has the greatest success of continued recovery*.*

## General conclusion

We show significant variation in three reproductive steroid hormones in female humpback whales and show evidence consistent with a temporal window in which this population experiences higher rates of estrous initiation. The field of conservation endocrinology is still rapidly evolving for wild marine mammals and will continue to provide novel and critical reproductive knowledge that will further assist in the conservation and management of these highly mobile and cryptic species. Humpback whales are sentinel species of ecosystem health, and changes in reproductive rates and health can provide quantifiable signals of the impact of environmental and anthropogenic change at the population level. This study was fundamental in its methodological approach to a wild species with a global distribution and seasonally distinct life histories. This information will assist in monitoring, managing and conserving this population as global ecological changes continue to occur. Further, this study supports the continued use of remote biopsy sampling and the development of long-term ecological research programs, which are critical to understanding the reproductive physiology and population dynamics of long-lived, highly migratory species. With increasing human use of the world’s oceans and the impacts of environmental change becoming more severe, many populations of marine mammals are at increased risk of population decline. Having a more comprehensive understanding of how marine mammals reproduce will inform conservation managers as to what levels of mortality and disturbance populations can tolerate and ultimately help us to identify those populations most at risk for the future changes to come.

## Supplementary Material

Web_Material_coae038
