# Supplementary material for: Demographic and physiological signals of reproductive events in humpback whales on a southwest pacific breeding ground
Source: Conserv Physiol. 2024 Jun 18;12(1):coae038. doi: 10.1093/conphys/coae038 (PMC11184982; doi:10.1093/conphys/coae038)
Supplement: Web_Material_coae038 [file web_material_coae038.zip › Supplemental_Material_Revision_1-CLEAN (1).pdf]

Supplemental Material

Methods:

Individual whales were identified using either a photograph of the underside of the fluke, the shape of the dorsal fin or scars present on the body, or by using a standard DNA profile. The DNA profile included sex-specific markers and microsatellite genotypes. DNA was extracted from the skin-blubber interface using either a using a proteinase K digestion followed by a standard phenol–chloroform extraction (Sambrook *et al.*, 1989) or from a commercially available kit (DNeasy 96 Blood & Tissue Kit, Qiagen, Hilden, Germany). The sex of each sampled whale was determined by amplification of sex-specific markers following the protocols of Gilson et al. (Aasen *et al.*, 1990, Gilson *et al.*, 1998). Results were compared to controls for a known male and female using gel electrophoresis. Samples were genotyped using 14 previously published microsatellite loci to help resolve the individual identity of each sampled whale and remove potential duplicates (Table S1) (Baker *et al.*, 2013, Berube *et al.*, 2000, Garrigue *et al.*, 2004, Palsbøll *et al.*, 1997, Valsecchi *et al.*, 1996, Waldick *et al.*, 1999).

Alleles were sized and binned using the software program Genemapper v3.7 (Applied Biosystems). The total number of amplified loci for a given sample was considered as an added quality control threshold, with samples amplifying for less than 12 loci considered poor quality and repeated or removed from final dataset. Given the estimated probability of identity for these loci from previous studies (Constantine *et al.*, 2012, Pallin *et al.*, 2018), we assumed that samples matching at a minimum of 12 loci to be recaptures of the same individual. The expected probability of identity (P_{ID}; the probability that two individuals drawn at random from a population will have the same genotype by chance) for each locus was calculated in GenAlEx v6.5 (Peakall *et al.*, 2006). Cervus 3.0.7 (Kalinowski *et al.*, 2007) was used to compute the number of alleles (K), observed and expected heterozygosity (HO and HE), and the probability of identity for all individual matches.

Results:

Individual identification and sex

151 samples from female humpback whales were used in the genotype analysis to aid in individual identification. On average, 13.89 loci were successfully genotyped per individual. The average P_{ID} for any given combination of 7 loci ranged from 4.86×10^{-11} – 1.00×10^{-6}, consistent with previous studies (Table S1).

Annual variation in reproductive hormones

The mean progesterone concentration for all female humpback whales sampled in 2016 (n = 20), 2017 (n = 75), 2018 (n = 48), and 20190 (n =28), excluding within-year recaptures, were 5.36 ± 2.3, 8.27 ± 16.34, 6.53 ± 9.41, and 14.24 ± 23.26 ng/g, respectively (figure S1). These were not significantly different (r^2 = -0.006, $F_{3,167}$ = 0.686, p = 0.562). The mean testosterone concentration for all female humpback whales sampled in 2016 (n = 20), 2017 (n = 75), 2018 (n = 48), and 2019 (n =28), excluding within-year recaptures, were 1.73± 0.74, 0.93 ± 0.64, 0.7 ± 0.61, and 0.26 ± 0.23 ng/g, respectively (figure S1). These were significantly different (r^2 =

0.291, $F_{3,167} = 24.26$, $p < 0.001$. The mean 17β-estradiol concentrations for all female humpback whales sampled in 2016 (n = 20), 2017 (n = 75), 2018 (n = 48), and 2019 (n =28), excluding within year recaptures, were 4.00 ± 1.34, 2.41 ± 1.76, 2.29 ± 1.93, and 1.02 ± 0.89 ng/g, respectively (figure S1). These were significantly different ($r^2 = 0.169$, $F_{3,167} = 12.59$, $p < 0.001$). A post hoc multiple comparison analysis is depicted in figure S1.

Tables

Table S1. Summary of microsatellite loci used for individual identification of humpback whales (*Megaptera novaeangliae)* sampled along New Caledonia(Berube *et al.*, 2000, Palsbøll *et al.*, 1997, Valsecchi and Amos, 1996, Waldick *et al.*, 1999). The number of alleles observed (H_O) and expected (H_E) heterozygosity) was calculated using *Cervus 3.0.1.* The expected probability of identity (P_{ID}) of each locus was calculated with the program *GenAlEx v6.5*.

Locus	Source	Label	[mgCl₂] mM	Size range (bp)	No. of alleles	H_E	H_O	P_{ID}
EV1	Valsecchi & Amos (1996)	NED	4	123-129	4	0.500	0.412	0.313
Ev14	Valsecchi & Amos (1996)	VIC	2.5	125-143	9	0.734	0.742	0.107
EV21	Valsecchi & Amos (1996)	FAM	1.5	109-119	6	0.662	0.698	0.153
Ev37	Valsecchi & Amos (1996)	NED	3.5	192-230	19	0.923	0.900	0.012
EV94	Valsecchi & Amos (1996)	FAM	2.5	202-220	9	0.810	0.788	0.062
Ev96	Valsecchi & Amos (1996)	FAM	1.5	147-171	12	0.866	0.926	0.032
GATA28	Palsbøll *et al.* (1997)	NED	2.5	147-203	13	0.603	0.583	0.177
GATA417	Palsbøll *et al.* (1997)	FAM	2.5	187-274	21	0.910	0.901	0.016
GT211	Palsbøll *et al.* (1997)	FAM	2.5	100-120	10	0.846	0.881	0.043
GT23	Berube *et al.* (2000)	VIC	2.5	101-123	9	0.809	0.821	0.063
GT575	Berube *et al.* (2000)	FAM	1.5	137-177	14	0.815	0.767	0.055
rw31	Waldick *et al.* (1999)	FAM	1.5	106-122	8	0.672	0.707	0.144
rw4-10	Waldick *et al.* (1999)	VIC	2.5	190-214	12	0.819	0.874	0.054
rw48	Waldick *et al.* (1999)	NED	3	108-120	6	0.719	0.775	0.122

Figures

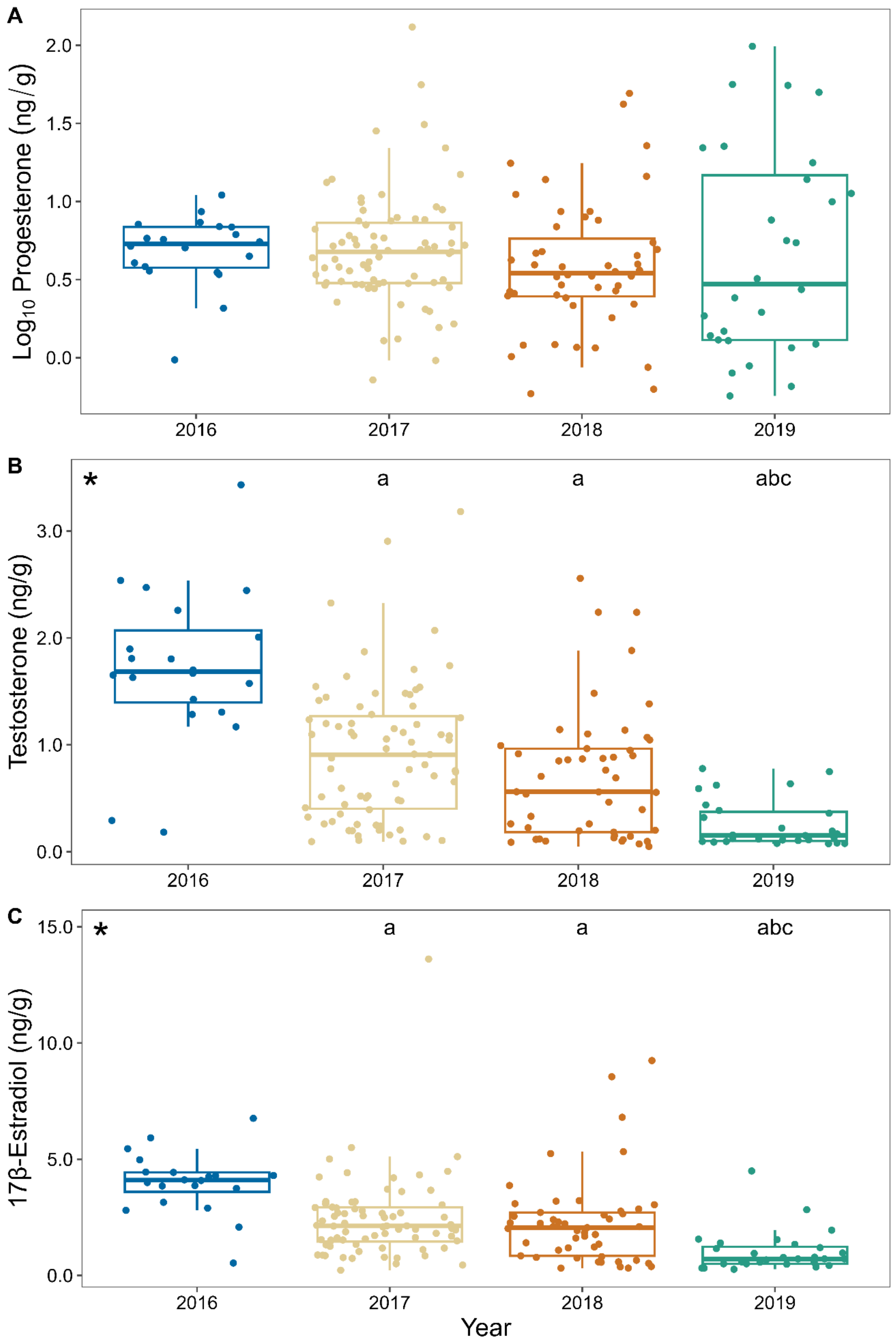
A
Log10 Progesterone (ng/g)
2.0
1.5
1.0
0.5
0.0
2016
2017
2018
2019
B
*
a
a
abc
Testosterone (ng/g)
3.0
2.0
1.0
0.0
2016
2017
2018
2019
C
*
a
a
abc
17β-Estradiol (ng/g)
15.0
10.0
5.0
0.0
2016
2017
2018
2019
Year

Figure S1. Yearly variation in female blubber progesterone (A), testosterone (B) and 17β-estradiol (C) concentrations (ng/g wet weight) of female humpback whales sampled in New Caledonia from 2016 to 2019. The asterisk (*) denotes a comparative statistically significant result via ANOVA. Post hoc multiple comparison analysis: a-significantly different from the year 2016; b-significantly different from the year 2017; c-significantly different from the year 2018.

Literature Cited

Aasen E, Medrano JF (1990) Amplification of the zfy and zfx genes for sex identification in humans, cattle, sheep and goats. *Bio/technology* 8: 1279-1281

Baker CS, Steel D, Calambokidis J, Falcone E, González-Peral U, Barlow J, Burdin AM, Clapham PJ, Ford JK, Gabriele CM (2013) Strong maternal fidelity and natal philopatry shape genetic structure in north pacific humpback whales:

Berube M, JØrgensen H, McEwing R, Palsbøll PJ (2000) Polymorphic di-nucleotide microsatellite loci isolated from the humpback whale, *megaptera novaeangliae*. *Molecular Ecology* 9: 2181-2183

Constantine R, Jackson JA, Steel D, Baker CS, Brooks L, Burns D, Clapham P, Hauser N, Madon B, Mattila D (2012) Abundance of humpback whales in oceania using photo-identification and microsatellite genotyping. *Marine Ecology Progress Series* 453: 249-261

Garrigue C, Dodemont R, Steel D, Baker C (2004) Organismal and 'gametic' capture-recapture using microsatellite genotyping confirm low abundance and reproductive autonomy of humpback whales on the wintering grounds of new caledonia. *Marine Ecology Progress Series* 274: 251-262

Gilson A, Syvanen M, Levine K, Banks J (1998) Deer gender determination by polymerase chain reaction. *California Fish and Game* 84: 159-169

Kalinowski ST, Taper ML, Marshall TC (2007) Revising how the computer program cervus accommodates genotyping error increases success in paternity assignment. *Molecular Ecology* 16: 1099-1106

Pallin LJ, Baker CS, Steel D, Kellar NM, Robbins J, Johnston DW, Nowacek DP, Read AJ, Friedlaender AS (2018) High pregnancy rates in humpback whales (*megaptera novaeangliae*) around the western antarctic peninsula, evidence of a rapidly growing population. *Royal Society Open Science* 5:

Palsbøll P, Bérubé M, Larsen A, Jørgensen H (1997) Primers for the amplification of tri-and tetramer microsatellite loci in baleen whales. *Molecular Ecology* 6: 893-895

Peakall R, Smouse PE (2006) Genalex 6: Genetic analysis in excel. Population genetic software for teaching and research. *Molecular ecology notes* 6: 288-295

Sambrook J, Fritsch EF, Maniatis T (1989) Molecular cloning. Cold spring harbor laboratory press New York.

Valsecchi E, Amos W (1996) Microsatellite markers for the study of cetacean populations. *Molecular Ecology* 5: 151-156

Waldick R, Brown M, White B (1999) Characterization and isolation of microsatellite loci from the endangered north atlantic right whale. *Molecular Ecology* 8: 1763-1765
